# Fractionated stereotactic radiotherapy for 136 patients with locally residual nasopharyngeal carcinoma

**DOI:** 10.1186/1748-717X-8-157

**Published:** 2013-06-27

**Authors:** Feng Liu, Jian-ping Xiao, Guo-zhen Xu, Li Gao, Ying-jie Xu, Ye Zhang, Xue-song Jiang, Jun-lin Yi, Jing-wei Luo, Xiao-dong Huang, Fu-kui Huan, Hao Fang, Bao Wan, Ye-xiong Li

**Affiliations:** 1 Department of Radiation Oncology, Cancer Institute and Hospital, Chinese Academy of Medical Sciences (CAMS) and Peking Union Medical College (PUMC), Beijing 100021, China

**Keywords:** Nasopharyngeal carcinoma, Residual lesions, Fractionated stereotactic radiotherapy, Boost irradiation, Toxicity

## Abstract

**Background:**

To evaluate the efficacy and toxicity of fractionated stereotactic radiotherapy (FSRT) in patients with residual nasopharyngeal carcinoma (NPC).

**Methods:**

From January 2000 to December 2009, 136 NPC patients with residual lesions after primary radiotherapy (RT) were treated by FSRT. The total dose of primary RT was 68.0-78.0 Gy (median, 70.0 Gy). The median time from the primary RT to FSRT was 24.5 days. Tumor volumes for FSRT ranged from 0.60 to 77.13 cm3 (median, 13.45 cm3). The total FSRT doses were 8.0-32.0Gy (median, 19.5 Gy) with 2.0-10.0 Gy per fraction.

**Results:**

Five-year local failure-free survival (LFFS), freedom from distant metastasis (FFDM), overall survival (OS), and disease free survival (DFS) rates for all patients were 92.5%, 77.0%, 76.2%, and 73.6%, respectively. No statistical significant differences were found in LFFS, DFS and OS in patients with stage I/II versus stage III/ IV diseases. Nineteen patients exhibited late toxicity. T stage at diagnosis was a significant prognostic factor for OS and DFS. Age was a prognostic factor for OS.

**Conclusion:**

FSRT after external beam radiotherapy provides excellent local control for patients with residual NPC. The incidence of severe late toxicity is low and acceptable. Further investigation of optimal fractionation regimens will facilitate reduction of long-term complications.

## Background

Nasopharyngeal carcinoma (NPC) is a radiosensitive neoplasm, and definitive radiotherapy (RT) remains the mainstay of treatment for NPC [1-7]. Although early-stage NPC is highly curative by radiotherapy alone, treatment results of loco regional advanced NPC remain disappointing. Local residual rate of NPC is about 10% and local recurrent rate ranges from 16.8% to 23%, depending on the initial tumor status [4-6]. Various methods have been used to improve local control (LC) of late stage NPC (UICC Stage III/IV), including concurrent chemo radiotherapy, high-dose IMRT with radio sensitization, and intracavitary brachytherapy [2,8-11]. The outcome of salvage treatment for local recurrence is poor, with a 5-year overall survival (OS) of 9.4–30%, and a high risk of complications [12-15]. Proper salvage radiation therapy is beneficial to achieve local control and improve survival of residual NPC, but it may increase therapy-related complications because of high doses of irradiation to normal tissues. Fractionated stereotactic radiotherapy (FSRT) or stereotactic radiosurgery (SRS) as a boost treatment for residual NPC is effective with improved local control and decreased complications. There were studies on using FSRT or SRS for local residual NPC, but the numbers of patients were relatively low or the follow-up time was limited [10,16]. Here we report our long-term outcomes and late toxicities in NPC patients who received FSRT for local residual disease.

## Methods

This study was approved by the ethics committee of Cancer Institute and Hospital, Chinese Academy of Medical Sciences and Peking Union Medical College.

### Patients’ characteristics

From January 2000 to December 2009, FSRT was administered to 136 NPC patients who had local residual lesions after the first course of external beam radiotherapy (EBRT). Pretreatment evaluation for all the patients included a complete history and physical examination, flexible fiberoptic nasopharyngolaryngoscopy, initial pathologic diagnosis, and multidisciplinary tumor board discussion. Staging evaluations included chest radiographs, complete blood count, complete metabolic panel, and MRI with gadolinium and/or CT with contrast from the base of skull to 2 cm below the clavicles. In selected patients with T3-T4 tumor or N2-N3 neck nodal disease, bone scans and chest and abdominal CT scans were also obtained.

Inclusion criteria for this study were as follows: pathological confirmation for all primary tumors prior to EBRT; existence of local residual tumor after EBRT and absence of distant metastasis. Residual tumor was defined as residual disease by fibroendoscopy or MRI within one week after completion of definitive EBRT. The criteria for residual disease on MRI were persistent tumor mass, thickened nasopharyngeal walls with enhancement at the primary site, or persistent enhancing retropharyngeal lymph nodes that were present before EBRT. If persistent lesion was observed by fibroendoscopy, tissue biopsy was not always necessary due to the risks of complications. If a superficial lesion in nasopharynx or adjacent area did not meet the diagnostic criteria of residual tumor on imaging studies, tissue biopsy is required within one week after imaging studies. Contraindications for biopsy were as follows: deep lesions that were located in parapharyngeal space, skull base, cavernous sinus or any areas that were not easily accessible; lesions abutting neurovascular tissues, evidence of infection or ulcer in nasopharynx, hemorrhagic tendency, or age older than 60 years or diabetes mellitus that cause healing delays. For the patients with suspicious residual lesions on imaging studies but tissue biopsy was contraindicated, we would repeat imaging studies at one month from completion of EBRT, using the same criteria for persistent disease described above. Residual Sites in this study included nasopharyngeal cavity, parapharyngeal space, retropharyngeal lymph nodes or intracranial residual lesions, while cervical nodes were not included. The patients with local residual tumor of NPC were evaluated jointly by a team of head-and-neck surgeons and clinical oncologists for possible salvage treatment. FSRT was recommended to these patients if it was considered the best available and acceptable treatment option. All patients were staged according to the 2002 International Union against Cancer (UICC) staging system. The twenty-five patients treated before 2002 were re-staged according to 2002 UICC staging system for the purpose of this study. Seventy-six patients (55.9%) had a single residual lesion, whereas 60 patients (44.1%) had multiple lesions, thus, a total of 196 lesions were treated by FSRT. All residual lesions were persistent in the high dose regions of EBRT. The main sites of local residual lesions were the pharyngonasal cavity, and the parapharyngeal space. Thirty-two (29%) patients also had skull base destruction. The details of the patient characteristics are shown in Table 1.

**Table 1:** Characteristics of NPC patients with residual lesions treated by FSRT

**Characteristic**	**Patients (%)**
Gender (n)	
Male	104 (76.5)
Female	32 (23.5)
Age (years)	Median 43 (range:13–77)
Stage *	
I	1 (0.7)
IIa	6 (4.4)
IIb	24 (17.6)
III	70 (51.5)
IVa	24(17.6)
IVb	11 (8.1)
Concurrent chemotherapy in primary RT	
No	61 (44.9)
Yes	75 (55.1)
EBRT	
Conventional EBRT	85 (62.5%)
IMRT	51 (37.5%)
EBRT dose (Gy)	Median 70 (range: 68.0–78.0)
Sites of local residual †	
pharyngonasal cavity	116 (85.3)
parapharyngeal space	53 (40.0)
retropharyngeal lymph nodes	26 (19.1)
cavernous sinus	1 (0.7)

* According to 2002 UICC staging system.

† Sixty patients had more than one site of residual disease.

### Primary external beam radiotherapy

In 85 patients, conventional EBRT was delivered at 2 Gy daily fractions, 5 fractions per week, to a median total dose of 70 Gy. The beam arrangements consisted predominantly of opposed parallel lateral ports, and all fields were treated with 6 MV beam photon. The lower neck was treated with an appositional anterior cervical field to 50 Gy. A block was used to protect the spinal cord at the field junctions for all patients. Fifty-one patients received intensity-modulated radiotherapy (IMRT) as the primary treatment of NPC. The gross tumor vo lume (GTV) with margin for daily positioning uncertainty was treated with 2.12-2.24 Gy fractions to a median total of 73.92 Gy (range, 69.96-78.0 Gy). The clinical target volume (CTV) at high risk for microscopic tumor involvement received 60.06 Gy in 30 fractions, and the low-risk CTV received 50.96 Gy in 30 fractions. All EBRT doses were limited to ≤40 Gy to the spinal cord dose, ≤54 Gy to the optic apparatus, and ≤54 Gy to the surface of the brainstem. The median overall time for EBRT was 50 days (range, 46–68). Seventy-five (71.4%) out of 105 patients with stage III/IV received cisplatin-based concurrent chemoradiotherapy. Thirty patients with stage III/IV cancer did not receive chemotherapy because of poor tolerance (such as severe comorbidities, low Karnofsky performance score) or being treated with RT alone on an institutionally randomized clinical trial.

### FSRT technique and treatment

FSRT was performed using a collimator-based system (Creat Stereotactic treatment system, China) with a 6-MV linear accelerator (Varian, USA) before July 2006, and multileaf collimator (MLC)-based FSRT system (BrainLAB, Germany) since August 2006. Patients were immobilized using a relocatable-type head ring with a wrapping head-band and stereotactic frame. Contrast-enhanced CT scan with a slice thickness of 3 mm was performed for treatment planning. If needed, MRI images were used to aid target delineation. The target volume was defined as abnormal soft-tissue mass or/and contrast-enhanced areas as shown in axial images plus a 2–3 mm margin. Adjacent mucosal and soft tissues were included if residual lesions were seen on flexible nasopharyngoscopy exam. Critical structures including brainstem, spinal cord, optic chiasm, optic nerves, eyeballs, and lenses were contoured.

**Figure 1:**
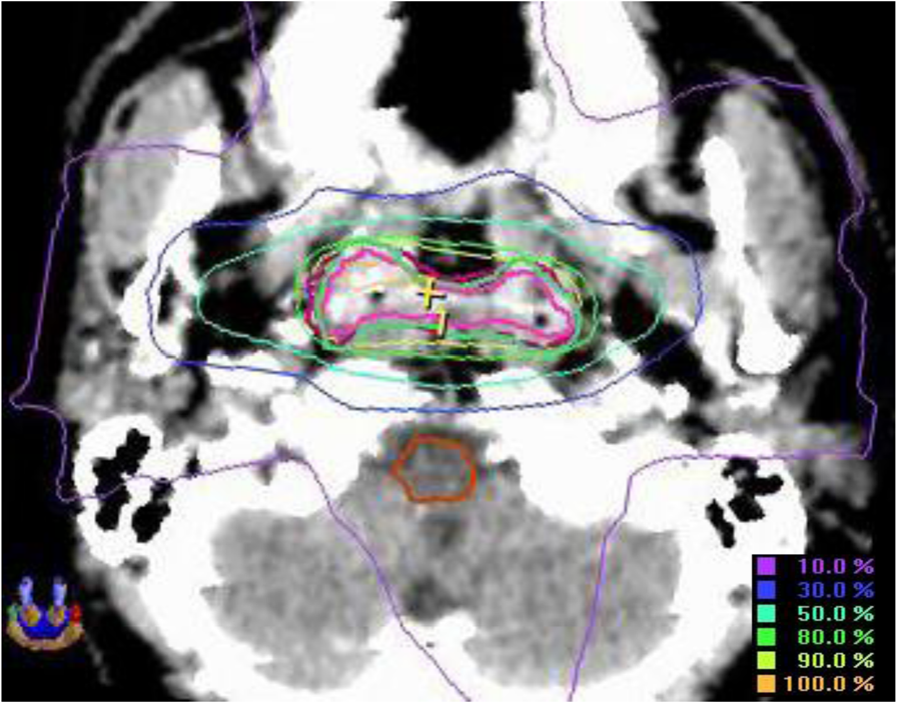
Isodose distribution using FSRT for residual disease at nasopharynx.

The fractional dose of FSRT was 2.0-10.0 Gy (median, 4.0 Gy) per fraction. The number of isocenters ranged from 1 to 3 (median, 1). Thirty-three patients received FSRT with fractionated dose>5 Gy, including 16-20Gy/8-10Gy/2f for 3 patients, 20-26Gy/6-8Gy/3-4f for 5 patients, 18-26Gy/5.2-7Gy/3-5f for 25 patients; and 103 patients received FSRT with fractionated dose≤5 Gy, including 12-32Gy/4-5Gy/3-8f for 49 patients, 8-23Gy/2-3.5Gy/3-7f for 54 patients. An example of isodose distribution is shown in Figure 1. To compare various fractionation schemes, the biologically effective dose (BED) was calculated using the equation BED=total dose×(1+d/α/β), where d=dose per fraction and α/β=10, no correction was made for tumor proliferation since all the treatment was finished within 2 weeks [17,18]. The median BED on FSRT was 28 Gy (range, 10.1-44.8 Gy). Table 2 summarizes the FSRT treatment parameters.

**Table 2:** Summary of FSRT parameters

**Parameters**	
Tumor volume (cm^3^)	13.45 (0.60–77.13)
Total prescribed dose (Gy)	19.5 (8.0–32.0)
Fractional dose (Gy)	
>5 Gy	33 (24.3)
≤5 Gy	103 (75.7)
Fraction number	5 (2–8)
Time to FSRT after EBRT (days)	24.5 (2–147)
FSRT type	
Collimator based	105 (77.2)
MLC based	31 (22.8)
Prescribed isodose line (%)	90 (70–90)
Number of isocenters	
1	82 (60.3)
2	46(33.8)
3	8 (5.9)

Abbreviations: Values are median (range) or number (percentage).

### Follow-up after FSRT

All patients were followed in our clinic every 3 months for the first 2 years, every 6 months for 3–5 years, and annually thereafter. MRI of nasopharynx and neck, and flexible nasopharyngoscopy were routinely performed during follow-up visits, and suspected lesion was subject to a biopsy. Blood chemistry panels, serum chemistries, chest radiographs, ultrasounds of abdomen, and bone scans were performed annually or if clinically indicated.

### End points and statistical analysis

The following endpoints were examined: response to FSRT, local failure-free survival (LFFS), overall survival, disease free survival (DFS), and freedom from distant metastasis (FFDM). Treatment response was evaluated by fibroendoscopy and MRI scans at six months after completion of FSRT. Treatment response was evaluated by imaging studies in most patients. If imaging studies were nondiagnostic, tissue biopsy would be mandatory if it was feasible. Complete response (CR) was defined as complete regression of tumor assessed on MRI and nasopharyngoscopy or a negative biopsy in patients with suspected lesions on imaging studies. Partial response (PR) was defined as tumor regression more than 50% of bi-dimensional diameters compared to lesions on baseline images, and no response if neither PR nor CR was achieved. In-field recurrence was defined as tumor relapse within the volume encompassed by FSRT prescription isodose line, and out-of-field recurrence as tumor relapse outside FSRT target volume but within the primary EBRT fields. Time was measured from the date when FSRT completed until time of event occurrence, or most recent follow-up for censored observations. Actual incidences of LFFS, OS, DFS and FFDM were calculated using the Kaplan-Meier method, and the differences between survival curves were compared using the log–rank test. Log-rank analysis was used to compare prognostic factors for LFFS, OS, DFS and FFDM, and factors found to influence prognosis on univariate analysis were subjected to multivariate analysis using Cox’s proportional hazard regression model, in order to determine if these factors acted independently. According to previous literature [19-22], gender, age(≤45 years vs. >45 years), interval between primary radiotherapy and FSRT(≤2 months vs. >2 months), T stage (T1–2 vs. T3–4) and N stage (N0–1 vs. N2–3) at diagnosis, tumor volume(≤10mL vs. >10 mL), BED(≤30 Gy vs. >30 Gy) and concurrent chemotherapy use during primary RT were analyzed as covariates in univariate and multivariate analyses. All statistical analysis was performed using the commercial software package SPSS 15.0 (SPSS Institute Inc., Chicago, USA). All acute and late complications were scored according to the Common Terminology Criteria (CTC) for Adverse Events v3.0 criteria and Radiation Therapy Oncology Group (RTOG) criteria.

## Results

### Treatment results

The median follow-up for all patients was 66.5 months (range, 10–139). The median follow-up for patients who were alive was 69 months (range, 20–139). At six months after completion of FSRT, the actuarial CR rate was 72.1% and PR rate was 23.5%, with an overall response rate of 95.6%.

**Figure 2:**
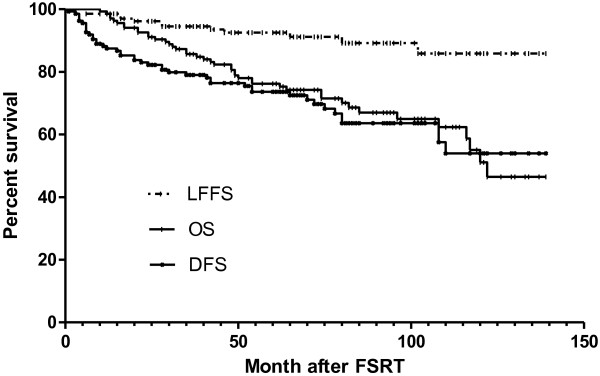
Local failure-free survival, overall survival and disease free survival after FSRT.

**Figure 3:**
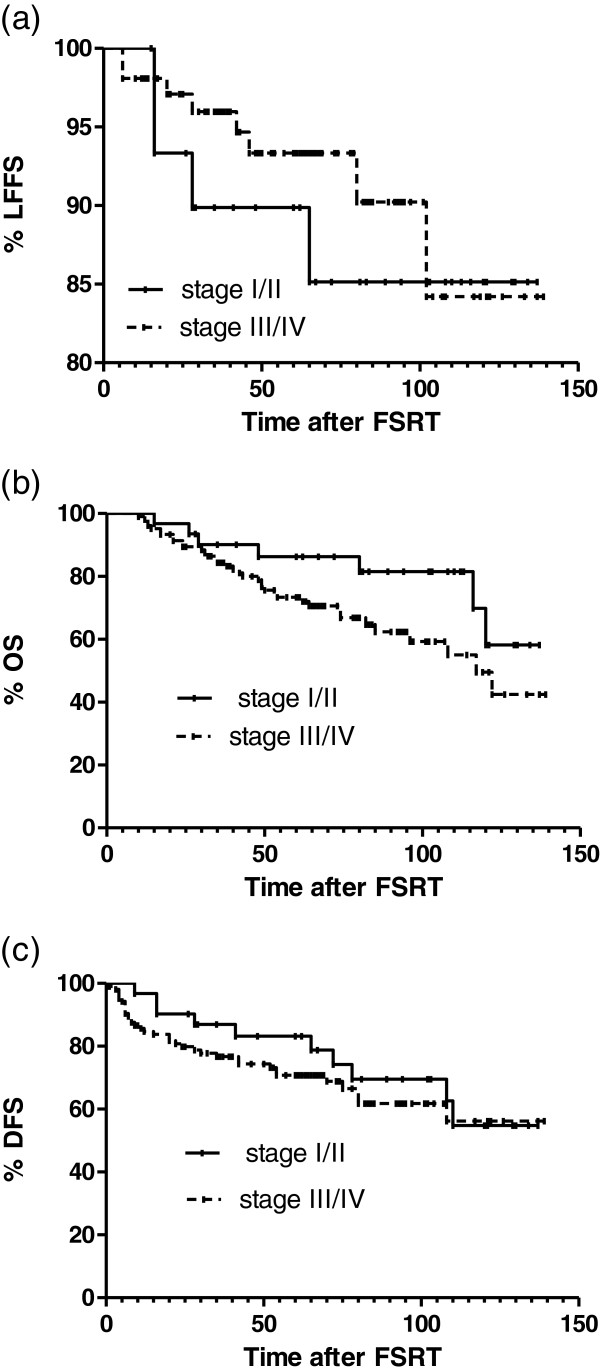
LFFS (a), OS (b) and DFS (c) after FSRT according to UICC stage.

Three- and 5-year LFFS and FFDM rates for all patients were 94.5 %, 92.5%, and 84.3 %, 77.0%, respectively (Figure 2). Three- and 5-year OS and DFS rates for all patients were 85.7 %, 76.2%, and 79.0 %, 73.6%, respectively. During follow-up, local relapse developed in 12 patients: 4 were in-field that occurred in 16–28 months, and 8 were out-field that occurred in 42–102 months after FSRT. Five patients developed cervical nodal recurrences, and they were salvaged by neck dissection and were nodal disease free since after. The median local relapse time after FSRT was 35 months (range, 16–102). Distant metastases (DM) were observed in 36 patients (26.5%) with a median time of 9 months (range, 1–110 months). The most common sites were the bones (24 patients), lungs (14 patients), and liver (8 patients). Ten patients developed more than one metastatic site. Of the patients who developed distant metastases, 6 had initial Stage IIb, 18 had Stage III, and 12 had Stage IV disease. Four patients had N0, 12 had N1, 15 had N2, and 5 had N3 disease. One patient had T1, 11 had T2, 15 had T3, 9 had T4 disease. For stage I/II and stage III/IV patients, 5-year LFFS, OS and DFS rates were 89.9% and 93.3% (p=0.577), 86.3% and 70.7% (p=0.0.097), 83.2% and 70.8% (p= 0.455), respectively (Figure 3). The cause of death was distant metastases in 22 patients, local recurrence with dyscrasia in 6 patients, postoperative severe dysphagia and dyscrasia in 1 patient, massive nasopharyngeal hemorrhage in 5 patients, other causes in 12 patients (including dyscrasia, cerebral hemorrhage and non-radiation induced pneumonia).

### Prognostic factors

Univariate analyses revealed that age (80.5% for patients ≤45 years vs. 70.7% for patients >45 years, p = 0.042) was a significant prognostic factor to predict 5-year actuarial overall survival, and T stage at diagnosis was significant for 5-year actuarial overall survival (83.9% for T1–2 vs. 70.1% for T3–4, p = 0.039) and disease free survival (86.2% for T1–2 vs. 63.6% for T3–4, p =0.039). No other variables were found to be significant prognostic factors for OS, DFS, LFFS and FFDM. The results of univariate analyses of OS and DFS are summarized on Table 3. Multivariate analyses showed that age and T stage at diagnosis were the significant independent prognostic factors for 5-year overall survival (p = 0.034 and p = 0.033, respectively), and T stage at diagnosis was significant independent prognostic factors for 5-year disease free survival (p = 0.043). The results of multivariate analysis are summarized on Table 4.

**Table 3:** Univariate analysis of prognostic factors for OS and DFS

**Parameters**	**5y-OS%**	**5y-DFS%**
Gender		
Male	73.6	73.9
Female	86.0	72.4
	p=0.276	p=0.50
Age		
≤45 years	80.5	72.1
>45 years	70.7	75.3
	p=0.042	p=0.848
Time interval from		
first radiotherapy		
≤2 months	78.5	74.7
>2 months	49.7	59.3
	p=0.40	p=0.925
T stage at diagnosis		
T1-2	83.9	86.2
T3-4	70.1	63.6
	p=0.039	p=0.039
N stage at diagnosis		
N0-1	80.8	75.2
N2-3	71.1	71.6
	p=0.195	p=0.805
Tumor volume		
≤10cc	81.3	78.5
>10cc	73.7	70.6
	p=0.173	p=0.260
BED		
≤30 Gy	80.3	73.8
>30 Gy	68.9	72.9
	p=0.415	p=0.968
Chemotherapy in primary RT		
no	78.7	75.0
yes	61.3	67.2
	p=0.316	p=0.317

**Table 4:** Significant prognostic factors on multivariate analysis

**Covariate**	**B**	**HR**	**P value**	**95% CI**
Overall survival				
Age	0.655	1.926	0.034	1.049-3.536
T stage at diagnosis	0.698	2.009	0.033	1.058-3.814
Disease free survival				
T stage at diagnosis	0.646	1.907	0.043	1.019-3.570

*CI* confidence interval, *HR* hazard ratio.

### Toxicity

There were no severe acute toxicities secondary to FSRT. All 136 patients completed the full course of FSRT as planned and treatment was well tolerated. Late toxicities included cranial nerve injury (IX-XII, grade 1–2) in 8, massive nasopharyngeal hemorrhage in 5, and asymptomatic temporal lobe necrosis in 6 patients. The diagnosis of temporal necrosis was all made according to magnetic resonance spectroscopy. Temporal lobe necrosis did not affect their job performance or daily life in these patients.

## Discussion

In 2006, we reported a 10-year OS of 66.5% for NPC patients [23], which was much higher than the earlier historical data reported by Qin et al. (5-year OS: 47.9%) [24]. One important reason to explain the improving treatment outcome is the dose escalation by boosting additional radiation to the residual primary lesions. Although some residual lesion might resolve without boost dose radiation, patients with residual NPC had a higher risk of local recurrence, and therefore additional doses for these patients have improved LC and OS [25,26]. Several other studies demonstrated that there was a dose–response relationship of NPC above tumoricidal levels [27,28]. As such, the boost radiation has become a standard salvage treatment modality for residual NPC in our institution.

One of the approaches to boost radiation dose for residual tumor is FSRT. A number of retrospective studies on using SRS or FSRT after primary EBRT for residual NPC have been reported, but the numbers of patients were small or the follow-up time was short (Table 5). A review of these reports suggests that FSRT improves the LC and OS of NPC with local residual lesions [19,20,29-34]. Chua et al. [35] treated 48 local failure NPC patients using SRS with a median dose of 12.5 Gy, and the 5-year LFFS and OS rate were 47.2% and 46.9%, respectively. Dhanachai et al. [21] treated 32 patients with residual or recurrent NPC using FSRT at a total dose of 17 to 59.4 Gy (4 to 25 fractions), the 3-year local progression-free rate and OS rate were 37.9% and 71.2%, respectively. Chen et al. [36] treated 64 patients with FSRT of 12–15 Gy, and the 3-year LC and OS rate were 93.1% and 84.9%, respectively. Our study demonstrated excellent LC and survival in the patients treated with SFRT for residual disease. Interestingly, with using FSRT for residual tumors, no statistical significance can be observed in LFFS, DFS and OS in patients with stage I/II versus stage III/IV disease.

**Table 5:** Clinical outcomes of SRS/FSRT for local persistent NPC in the literature

**Author**	**Number of cases**	**Modalities**	**Dose**	**Results**	**Major complications**
Chang et al. (1999) [29]	23 (P)	SRS	7-15 Gy	2-y LC 100%	no
Tate et al. (1999) [30]	23 (P)	SRS	7-15 Gy	2-y LC 100%	no
Ahn et al. (2000) [31]	19 (B)	FSRT	8-40 Gy/4-20 f	4-yr LC 89%	LMN 1 (5.3%)
				4-yr OS 75%	
Xiao et al. (2001) [32]	32 (P)	FSRT	14-24 Gy/2-4 f	3-y DFS 74%	FNH 2 (6%)
				3-yr OS 70%	
Le et al. (2003) [19]	45 (P)	SRS	7-15 Gy	3-y LC 100%	CNI 4 (8.9%)
				3-y OS 75%	TLN 3 (6.7%)
Chua et al. (2003) [33]	7 (P)	SRS	11-14 Gy	2-y LC 100%	no
Wu et al. (2007) [20]	34 (P)	FSRT	18 Gy/3 f	3-y LC 89.4%	TLN 3 (8.8%)
				3-y DFS 80.7%	
Hara et al. [33] (2008)	82 (B)	SRS	7-15 Gy	5-y OS 69%	TLN 10 (12.2%)
Present study	136 (P)	FSRT	8-32 Gy/2-8 f	5-y DFS 73.6%	CNI 8 (5.9%)
				5-y OS 76.2%	TLN 6 (4.4%)
					FNH 5 (3.7%)

*P* persistent lesions, *B* boost, *LMN* local mucosal necrosis, *FNH* fetal nasopharyngeal hemorrhage, *CNI* cranial nerve injury, *TLN* temporal lobe necrosis.

Analysis of our data helped us optimize FSRT regimens for residual NPC. Before April 2006, cranial nerve injury and massive nasopharyngeal hemorrhage were observed in 8 and 5 patients. Since August 2006, we cautiously adjusted the FSRT regimens. To avoid severe neurovascular complications, the guidelines of lower total dose of 10–21 Gy and fractional dose of 2.5-4 Gy (with a BED ≤27.3 Gy) were used with following indications: small tumor volume (≤30 cm^3^), residual tumors abutting carotid sheath or invading pharyngeal recess, cavernous sinus or foramen lacerum, IMRT as primary RT, short interval between primary RT and FSRT (≤2 months), age<15 or>70, and concurrent chemotherapy during previous EBRT. For rest of patients who did have any features mentioned above, we tended to deliver relatively higher total dose of 15–24 Gy (3–4 Gy per fraction with BED ≤33.6 Gy) in order to achieve satisfactory tumor control. The indications for high dose were larger tumor volume (>30 cm^3^), residual tumors not abutting carotid sheath, pharyngeal recess, cavernous sinus or foramen lacerum. Other indications for higher total dose included conventional EBRT, relatively long interval between primary RT and FSRT (>2 months), age 15 to 70, and those who did not receive concurrent chemotherapy.

On analyzing the prognostic factors, we found that T stage at diagnosis was a significant independent predictor of OS and DFS, age was significant prognostic factor for OS. Our results are consistent with previous studies [19,22]. Chua et al. [22] reported that T stage, tumor volume and time interval form primary radiotherapy were significant predictive factors of LC and survival whereas age was of marginal significance in predicting survival. On the other hand, Le et al. [19] reported that age was significant prognostic factor for survival. Our data showed no benefit of adding chemotherapy in NPC patients who received FSRT, which is in concordance with Le et al.’s and Wu et al.’s study [19,20]. In this retrospective study, only advanced stage NPC were treated with chemotherapy but not early stage disease, so the entire group disease local control and survival benefits from chemotherapy might have been reduced or weakened. To address the role of chemotherapy in patients who received additional FSRT, a phase III randomized study should be conducted.

Dhanachai et al. [21] reported significantly worse LC in patients receiving chemotherapy, but the author stated that it was not possible to conclude that chemotherapy led to the poor LC because there were selection factors which could lead to the worse outcome. In our study, tumor volume was not a significant independent predictor. The prognostic impact of tumor volume was not concordant with previous studies. Chua et al. [22] reported significant association between tumor volume and LC or survival. Wu et al. [20] reported that tumor volume was a significant predictor of progression-free survival (PFS) and distant metastasis-free survival (DMFS) but not LFFS, While Dhanachai et al. [21] reported that chemotherapy was the only significant factor for local PFS.

Radiation-induced nasopharyngeal necrosis and massive hemorrhage are consequential late effects in patients with NPC [37]. The mortality reported for massive nasopharyngeal hemorrhage in residual or recurrent NPC after FSRT is from 16.0% to 22.2% [20,21,32,35].

In 2001, we reported 8 of 50 patients died of fatal hemorrhage after FSRT [32], and thereafter, we reduced the fractionated dose. In this study, 5 patients developed massive nasopharyngeal hemorrhage, but two of them were treated in 2000 to a total dose of 23–25 Gy at 5.0-5.75 Gy per fraction. The 3 other patients were treated with FSRT between August 2004 and April 2006, to a total dose of 14–16 Gy at 3.5-4.0 Gy per fraction. These 3 patients had primary tumor abutting the carotid artery, combined with nasopharyngeal infection and ulceration. Massive nasopharyngeal hemorrhage has not been observed since we carefully chose lower fractionated dose (≤3 Gy per fraction) in patients who had high risk hemorrhage factors, controlled nasopharyngeal infection, and support patients’ nutrition. Other factors predicting potential risk of fatal hemorrhage include high cumulative dose of FSRT and EBRT, previous nasopharyngeal brachytherapy and nasopharyngeal necrosis or infection. Preventive management for nasopharyngeal necrosis that was used in our study included daily nasopharynx douche, nasal oily drops and epithelial growth factor for healing the ulceration.

Before 2006, 8 patients were observed symptomatic cranial nerve injuries (5.9%), including 5 hypoglossal nerve injury (3.7%), and 3 unilateral vocal cord paralyses (2.2%).

The FSRT regimens included 24 Gy in 4 fractions (3 patients), 18 Gy in 3 fractions (4 patients), 19.5 Gy in 3 fractions (1 patient). All cranial nerve injuries were on the same side of the residual lesions that were treated with FSRT. These patients had residual tumor involving parapharyngeal space or abutting the carotid artery, it was difficult for FSRT to completely avoid neurovascular bundles. In addition, higher fractional dose (≥6Gy) could be a factor causing cranial nerve injury. Since August 2006, we used revolving conformal MLC-based FSRT, with reduced fractionated dose (≤4 Gy) and low irradiation dose to carotid sheath (less than 2 Gy per fraction) for patients with high risk of cranial nerve injury. We have observed 6 patients (4.4%) developed temporal lobe necrosis following FSRT, which was probably secondary to primary conventional radiotherapy; and the FSRT dose delivered to temporal lobe on these patients was relatively low. In our experience, FSRT did not exceed expected mucous membrane toxicity, no more than grade 2 toxicities had been observed. With the increasing use of dose escalation, and improvements in disease local control and long-term survival, avoiding potential late neurovascular injuries has become a priority.

Since the completion of the Intergroup 0099 trial, which demonstrated improved LC control and survival with concurrent chemoradiotherapy, this has become a widely accepted treatment for patients with locoregional advanced NPC [38]. Although the use of cisplatin-based concurrent chemoradiotherapy was associated with a higher LC, many still failed distantly, and survival becomes highly dependent on distant control. There were 12 (8.8%) patients in our study that developed local failure after FSRT, while DM was still the major relapse form in this group. Wu et al. [20] reported that the DMFS rate was 80.8% and 67.2% respectively in residual and recurrent patients after FSRT. Twenty-two patients (25.3%) developed DM 3 to 8 months after FSRT, with relapse of nasopharynx or local lymph nodes in 5 patients. Le et al. [19] reported a 3-year distant-metastasis rate of 31.1% (14/45) after SRS. Hara W et al. [34] treated 82 NPC patients (57% stage IV) using SRS boost after 66Gy of EBRT, and the 5-year FFDM rate was 68%. In our study, the 3-year FFDM rate was similar to Wu et al’s results, while the 5-year FFDM rate was higher than Hara W et al’s results, probably attributing to the higher EBRT dose and less proportion of patients in stage IV in our study. With improved LC, the predominant site of failure is distant, and more effective systemic treatment is needed in these patients.

Based on our experience, a total dose of 15 Gy at 3 Gy per fraction seems to be optimal for satisfactory tumor local control and reduce the risk of severe neurovascular complications. For patients with small tumor volume (≤30 cm^3^), short interval between primary RT and FSRT (≤2 months), primary IMRT, or high risk complication features (residual tumor abutting neurovascular structures, age<15 or >70, concurrent chemotherapy during previous RT), we recommended FSRT with a total dose of 12 Gy at 3 Gy per fraction. Relatively higher total dose has been used in our practice for those with large tumor volume (>30 cm3), long interval between primary RT and FSRT (>3months), primary conventional EBRT, no risk complication features. The recommended FSRT regimen for those patients was generally 12–15 Gy (no more than 21) at 3 Gy per fraction.

## Conclusion

In summary, our results indicated that FSRT following definitive RT or chemoradiation is an effective treatment modality for patients with residual NPC. This study reiterates that FSRT provided satisfactory tumor control and DFS with acceptable toxicities. Distant metastases still represent the major causes of failure. Although our study was limited by the retrospective nature, the outcomes are encouraging, and further prospective randomized study should be conducted to better define the role of treatment outcome and optimal FSRT fractionations.

## Consent

Written informed consent was obtained from the patient for the publication of this report and any accompanying images.

## Competing interests

The authors declare that they have no competing interests.

## Authors’ contributions

J-PX and G-ZX carried out the study of fractionated stereotactic radiotherapy (J-PX is the corresponding author, and G-ZX is the co-corresponding author). FL, YZ and X-SJ were in charge of patients’ treatment. FL analyzed the data and drafted the manuscript. Y-JX, F-KH, HF and BW executed the fractionated stereotactic radiotherapy as technicians. Y-XL, LG, J-LY, J-WL and X-DH helped to revise the manuscript. All authors read and approved the final manuscript.
